# Geriatric mental health: Recent trends in molecular neuroscience

**DOI:** 10.4103/0019-5545.58886

**Published:** 2010

**Authors:** T. S. Sathyanarayana Rao, B. Praveena, K.S. Jagannatha Rao

**Affiliations:** Department of Psychiatry, JSS University, JSS Medical College Hospital, Ramanuja Road, Mysore - 570 004, India; 1 Department Biochemistry and Nutrition, CFTRI, Mysore-570 020, India

For every eight seconds, a Baby Boomer turns 60 in the world. By 2015, nearly 15% of our population will be over 65 years of age. This is increasing and expected to increase geriatric population burden in health and financial sector in every country. We have limited geriatric healthcare and care giving facilities and this is the essential need due to changing social and financial spheres in our lives. Research on aging and aging processes leads the way to a greater understanding of all age-related diseases.[1–6] It has the potential to improve public health to a far greater extent than science that examines only one disease at a time. Research on aging also provides the hope and the promise for everyone to live healthier, longer lives, less susceptible to disease and disability. Aging research is likely to be the least expensive path to preventing and curing many diseases of aging. Studying aging requires two complementary approaches: studying the components of disease that are related to aging and studying the underlying mechanisms of aging and how they regulate the processes in our bodies.[7,8] In USA, for 28 years, the American Federation for Aging Research (AFAR) has been at the forefront of this revolutionary approach to the science of healthier aging. AFAR has played a major role in providing and advancing knowledge of aging and mechanisms of age-related diseases by providing grants to more than 2,600 talented scientists. There is an urgent need in India too to have advanced research in aging related to Indian situation. We don't have any cross sectional study either on healthy aging or age related neurological disorders and their burden in India. Government of India need to play a significant role in funding geriatric research.

Americans are living longer than ever, according to the latest life expectancy statistics. But American men still aren't living as long as American women. The average life expectancy for men in the US is now roughly 75 years. For women, it's more than 80. Exactly why men are shorter-lived than women isn't entirely clear. Even in infancy, boys run a higher risk of dying than girls, and researchers aren't sure why. Research, however, does suggest that a leading reason for the “longevity gap” between men and women is that men don't take care of themselves as well as women do. Surveys have found, for example, that women are much more likely to have a regular healthcare provider, and to see him or her within the course of the year, than men are. Men are also more likely to engage in “risky” behavior - like smoking and drinking heavily then women.

The greatest health asset for geriatric population is spending time and doing things with other people, of all ages, which can help keep them mentally, physically and emotionally fit. It can also give brain a boost and lift the mood. They need to volunteer, or join community or other groups and get involved in activities they enjoy. But in the current situation is not so satisfactory for our aged. In India, there is an urgent need to develop models to keep geriatric populations active through out life. We need to provide a comprehensive plan on post retirement healthy and active living.[9–11]

Unhealthy aging is a risk factor for geriatric mental disorders. The failure in normal healthy aging leads to mental disorders in aged population. Bipolar disorder (BD) is a major geriatric mental health problem and affects about 1% of the population and causes severe neuropsychological impairments and is implicated in functional impairment.

What is meant by normal aging and healthy aging and what are the triggering risk factors for geriatric mental health problems are the major puzzling issues. We need to understand biology of aging properly. Biological changes during aging include neuropeptide (involved in memory and emotion), calcium balance, hormones like oxytocin, neural networks, metal homeostasis, and genetic instability and related gene expression changes. It is worth looking into some of these aspects which has drawn the attention of researchers.

Depression is a common neuropsychiatric syndrome in neurodegenerative disorders (ND). However, it is not clear whether changes in neurotransmitter activities lead to a specific profile of depressive symptoms in ND and further obscure on how it differs from that in depressed patients without ND. Ehrt *et al*.[12] reported that the magnitude of depression in ND patients is comparable with that of depressed control subjects. The clinical features indicate that patients with ND showed less sadness, anhedonia, feelings of guilt and less loss of energy, while these symptoms are more predominant in depressed control subjects. These findings are important for molecular understanding of neurochemical changes between normal aging, ND and clinical diagnosis in geriatric population.

Zhu *et al.*[13] reported that neurotrophin levels would be influenced by differential housing and their relation to emotional behavior in animal models. Environmental enrichment condition (EC) brings about changes in behavioral, neurochemical and neuroanatomical aspects. There are evidences to show that the hippocampus is one of the most susceptible brain areas to the effects of enriched rearing. Studies also indicate that the hippocampus is functionally segregated; and the dorsal hippocampus is important for spatial learning, while the ventral part of hippocampus is critical in emotional behavior. The above authors studied the influence of differential housing environment on anxiety-related behavior and neurotrophin levels in dorsal and ventral hippocampus, and other brain regions in animal models. Subsequently, brain nerve growth factor (NGF) and brain-derived neurotrophic factor (BDNF) were analyzed in selected brain regions of the tested and non-tested animals. Differential housing influenced anxiety-related behavior in the plus-maze and brain neurotrophins. They found that there are changes in baseline levels of BDNF and NGF protein in hippocampus due to environmental enrichment and have an impact on emotional behavior.

Mohammed *et al.*[14] reported that novel environment enrichment causes significant changes in the hippocampal and septal NGF levels. And their studies indicated that the lack of adequate environmental stimulation might be of importance in age-related behavioral and neurochemical deficits. This has great application in understanding geriatric mental health. A study by Nordberg[15] reported the role of human nicotinic receptors in aging and dementia. Normally, multiple nicotinic receptors seem to be present in brain as indicated by neurophysiologic, neurochemical, and molecular studies. However, their role in higher functions including learning and memory are still not clear. The changes in nicotinic receptor subtypes in human brain in normal aging is not clear, however there is decrease in brain nicotinic receptors in neurodegenerative brain. Now scientists are trying to develop new therapeutic compounds to increase cholinergic activity through the nicotinic receptors in mental illness. Recently our team (unpublished data) found new data on genomic instability and its correlation to redox metal imbalance in aging human brain. DNA conformation and stability play an important role in brain function. The earlier studies reported alterations in DNA integrity in the brain regions of neurological disorders like Parkinson and Alzheimer's diseases. However, there is limited study on DNA stability in aging brain and factors responsible for genomic instability still not clear. In this study, we assessed the levels of Copper (Cu), Iron (Fe) and Zinc (Zn) in three age groups (Group I: below 40 years), Group II: between 41-60 years) and Group III: above 60 years) in hippocampus and frontal cortex regions of normal brains. Genomic DNA was isolated and DNA integrity was studied by nick translation study and presented as single and double strand breaks. We observed that the levels of Cu and Fe are significantly elevated while Zn is significantly depleted from Group I to III with aging in frontal cortex and hippocampus regions. But the elevation of metals was more in Frontal cortex region compared to hippcampal region. The number of single strand breaks correspondingly increases with age compared to double strand breaks. The strand breaks were more in Frontal cortex compared to hippocampus. There is a clear correlation between Cu and Fe levels versus strand breaks in aging brain regions. This indicates that genomic instability is progressive with aging and this will alter the gene expressions. To our knowledge to date, this is a new comprehensive database on the levels of redox metals and corresponding strand breaks in DNA in two brain regions of human aging brain. These findings have a great biological significance with relevance to geriatric mental health and further work is in progress in this direction.

There is a great need to develop biomarkers between normally healthy geriatric population and mentally ill geriatric patients. The search for late-life mental disorders includes neuroimaging, cerebrospinal and serum biomarkers, biochemical neurotransmitters and metals.[16] Recently, there was a debate that serum homocysteine is a good biomarker to predict cognitive dysfunction. The study by Hengstermann *et al.*[17] investigated the relation between higher homocysteine levels versus cognitive dysfunction in multimorbid geriatric patients and found no relation. Still we don't have a good biomarker for any neurodegenerative or mental illness disorders, hence difficult to segregate between health geriatric and mentally ill geriatric individual.

There is great need to understand the link between neuropsychiatry to neurodegeneration through MRI application. There is wide scope for geriatric mental health research with molecular biology application and imaging tools. Also there is great need to establish biomarkers for healthy aging and this is an open area for research. There is lot of debate on genetic predisposition of mental disorders in aged population but genomic and proteomic approach to unravel the above phenomena.[18,19] Both structural, chemical, functional brain imaging using magnetic resonance image and post-mortem studies have demonstrated volume loss in brain in subjects with BD and aging. Recent post-mortem studies in BD also provided an evidence for reductions in number and density as well as changes in cell body size and shape of neurons and glia, implicating specific cell pathology in the mood disorders and control aged brains. All these studies give insight into neuronal cell death which may play a central role in the pathology of mental health and failure in normal healthy aging. The major risk factors attributed for age related disorders, is the elevation in oxidative stress and failure in antioxidant mechanisms. The oxidative stress phenomena leads to DNA instability and gene expression failure in normal aging and does the failure in repair mechanism lead to neuropsychiatry problems? The data base on this aspect is very limited due to lack of research in this dimension. A dysregulation in cell death mechanism is believed to play a role in a variety of neuropsychiatric disorders. Prolonged neuropsychiatry disorders are likely to be risk factors for neurodegenerative disorders. Apart from biology, there is an urgent need to develop geriatric specialty foods to meet the age related demand for nutrients and also bring about geriatric social clubs for healthy living together and also prefer aged population as paid volunteers in many sectors so that the late life is active both physically and mentally. In the West, this is a special provision, which is missing in India.

Nutrition plays an important role in health and it seems to be one of the major player of successful aging. The adequate nutrition has a significant role in a healthy lifestyle and contributes for good mental functioning. However, malnutrition makes susceptible for disease. As on present, recommended dietary allowance (RDA) is a guideline to compute the needs of populations of the healthy elderly. However, there is not enough research done on diet and geriatric health and mental illness. In the future, special recommendations for subgroups of individuals are essential, taking into account individual health status and genetic factors. Elderly food needs to be rich in complex carbohydrate sources, fruits and vegetables with fiber, antioxidant, and essential fatty acids. Great deal of research needs to be undertaken in this direction and attention to be paid for formulating geriatric specialty foods.

There is a need for major research on geriatric specialty foods in India; to develop knowledge base on healthy aging for 30 years above, to conduct research in static and dynamic life style versus aging, life style diseases in Geriatric population, geriatric mental health etc. We need to have special research programs in Indian Council of Medical Research and also attract private funding to develop data base on Geriatric health status in India. The research contributions of India in geriatric research is less than 1% in the world scientific literature and extensive research on geriatric mental health using social, biochemical, genetics and molecular aspects is and around the world[20–25] and Indian Government is spending less than 0.1% of GDP on geriatric health research and care. There is an urgent need for the right direction and India need to participate extensively in geriatric mental health research.
